# Public-House Trusts

**Published:** 1901-08-10

**Authors:** 


### PUBLIC-HOUSE TRUSTS.

As it seems that the time is still far off when the Legislature will undertake to deal with the question of the liquor traffic on a national basis, philanthropists are beginning to try to acquire the management of public-houses, with a view to, first, preventing that accumulation of wealth and corresponding power in the hands of individual proprietors which enables them, in many cases, to obtain an influence over local authorities which is contrary to the public interest, though eminently beneficial to the financial interests of the licence-holders; secondly, to ensure the expenditure of all profits derived from the sale of liquor, beyond a fair interest on the capital involved, on objects of public benefit, and especially on counter-attractions to the public-house; thirdly, to the provision of refreshments, alcoholic and non-alcoholic, which shall be of sound and wholesome quality. To this end "Public-House Trust" Companies have been formed in many places, and are in course of formation in more. Such companies exist already in Fifeshire, Glasgow, Renfrewshire, Northumberland, Kent, and Belfast, and preliminary steps to the formation of them have been taken in Bradford, Durham, Essex, Leeds, Liverpool, Northamptonshire, Nottinghamshire, Surrey, Sussex, Warwickshire, Hertfordshire, and Hampshire.

The idea of these companies is not altogether new, and has been already carried out by private individuals. We think The Hospital was the first, and for a long time the only journal to take notice of the experiment of the Rev. Osbert Mordaunt at Hampton Lucy, which succeeded at once in lessening the drinking habits of the village community over which, as squire and parson, he exercises influence, and in providing the villagers with a good water-supply, lights in the street, and other conveniences, all paid for out of the profits of the tavern. There need be no doubts as to the profits. When 5 per cent. has been paid on capital, when a sufficient sum has been put aside for depreciation and reserve, there is sure to remain a goodly sum, which now enriches private individuals, but might be used for the advantage of the community at large. The largest existing company of the kind is probably the "People's Refreshment House Association," of which the Bishop of Chester is president, and its social and financial success has been such as to encourage the formation of others. The capital of the company, whether large or small, is usually divided into a number of ordinary £1 shares purchasable by anyone, and a small number of deferred shares, of the nominal value of a shilling, which carry 50 per cent. of the voting power of the company, and are allotted exclusively to trustees who are expected to expend the whole of the net profits, after the payments indicated above have been made, on objects of public utility.

The rules of management in all licensed houses carried on by public-house trust companies are much the same. The manager is paid a fixed salary, but may have a commission on the sale of food and non-intoxicating liquors, which are to be as readily accessible as alcoholic drinks. He has no commission on the sale of intoxicants. All refreshments, whether food, alcoholic or non-alcoholic liquors, are to be of the best quality obtainable in the open market, and nothing approaching the "tied house" will be tolerated. The companies try to obtain licence for their public-houses in places where the population is so large as to render it likely that magistrates will grant them, they establish canteens and refreshment bars in the neighbourhood of large works, and purchase, rent, or manage existing public-houses. In two respects, however, the policy of the trusts differs from that of private licence-holders. If they are refused a licence in any place, they will not inevitably go away lamenting. Like philanthropic dogs-in-the-manger, they will rejoice that they have at least kept out a private licence-holder, who might carry on the business less conscientiously, for in very few places will magistrates, for the sake of their own repute, venture to give a licence to a private applicant and refuse it to the trust. And, in the second place, when they buy an existing public-house, it will not inevitably be for the sake of carrying it on, but for the sake of closing it, if, in the opinion of the directors, there are too many public-houses already in the neighbourhood. In short, all the efforts of the company will be directed to the diminution of drinking, not to its increase.

Notwithstanding the honourable aims of the promoters of these trusts, they have met with much unfavourable criticism. The extreme teetotallers, who object to the municipalisation of the liquor traffic, on the ground that "it will make us all partners in the sin of drunkenness," urge similar pleas against the trusts. They want prohibition, and are apparently unable to see that where prohibition is impossible, as it is in all large places, it is a good thing to have the control of the dangerous traffic in strong drink in the hands of honourable people who have no selfish aims to serve. It is useless to argue with these persons. But persons of less extreme views often hold aloof from these companies for fear that the very fact that the surplus profits are to be devoted to objects of public utility may make people inclined to condone drunkenness for the sake of the profits themselves. If the public library, or the public baths, or the street lighting, or, above all, if the rates profit from the money gained by the profits of the liquor traffic, will the public desire that that traffic should diminish?

We think that on this point the public conscience is less dull and selfish than these timorous folks believe, but in any case we think that the elimination of private profit is such a great good that some risks may be run to obtain it. It is the greatness of profit that makes the publican such a power as he is in many places. Nor, on the ground of public morality, is it to be forgotten that these big profits tempt men to go into the liquor trade who, if the profits were less big and less easily earned, would turn to a business less dangerous to the public welfare. Moreover, the profits earned by the trusts are largely devoted to counter-attractions to the public-house, which in time are bound to do good even though their direct attractiveness to a confirmed drunkard may not be apparent. But drunkenness is often the resort of the ignorant simply because they know of no other pleasure. As life widens out to them, as their desires increase, as they begin to want better houses, country holidays, books, theatres, concerts, they will take less drink because there will be other things which they want more. To break a man from drinking it is necessary to do more than keep drink from him. You must teach him to want something else.

